# Marine *Aspergillus*: A Treasure Trove of Antimicrobial Compounds

**DOI:** 10.3390/md21050277

**Published:** 2023-04-28

**Authors:** Honghua Li, Yanqi Fu, Fuhang Song

**Affiliations:** Key Laboratory of Geriatric Nutrition and Health, Ministry of Education of China, School of Light Industry, Beijing Technology and Business University, Beijing 100048, China; lihonghua@btbu.edu.cn (H.L.); fuyanqi0601@126.com (Y.F.)

**Keywords:** marine *Aspergillus*, secondary metabolites, antimicrobial activity

## Abstract

Secondary metabolites from marine organisms are diverse in structure and function. Marine *Aspergillus* is an important source of bioactive natural products. We reviewed the structures and antimicrobial activities of compounds isolated from different marine *Aspergillus* over the past two years (January 2021–March 2023). Ninety-eight compounds derived from *Aspergillus* species were described. The chemical diversity and antimicrobial activities of these metabolites will provide a large number of promising lead compounds for the development of antimicrobial agents.

## 1. Introduction

Compared with terrestrial fungi, marine fungi are more abundant in species. Due to the complex environment, their metabolites have novel structures and diverse activities [1,2,3,4]. As an important member of marine microorganisms, fungi play an important role in the study of active natural products. Marine fungi can be obtained from marine animals, plants, sediments and seawater [5,6,7,8]. Therefore, marine fungi have a wide range of sources [6,9,10,11,12,13,14,15].

*Aspergillus* is a genus of fungi widely distributed in marine environments [16,17,18]. Common species include *A. fumigatus*, *A. niger*, *A. versicolor*, *A. flavus*, *A. ochraceu*, *A. ticus*, *A. terreus*, etc. Marine *Aspergillus* is an important resource in the production of active natural products, such as steroids, flavonoids, azolones, etc. [7,19,20,21,22]. These metabolites are structurally diverse and exhibit a wide range of biological activities, including anticancer, antiviral, antibacterial, anti-inflammatory, lipid-lowering and anti-diabetic [22,23,24,25,26,27].

Due to the wide range of *Aspergillus* sources, the diverse secondary metabolites and the wide biological activities, the research on *Aspergillus* metabolites has attracted much attention. Therefore, a series of excellent reviews on this subject have been published so far [28,29,30,31,32,33,34,35,36,37,38,39]. In 2016, Fouillaud et al. reviewed the knowledge of anthraquinones and their derivatives derived from filamentous fungi [40]. In 2022, Hafez Ghoran et al. updated this study and summarized and classified the structures and activities of 296 anthraquinones and their derivatives [41]. In 2019, Youssef et al. reviewed the chemical and biological activities of peptides which isolated and identified from marine fungi [22]. 131 peptides were reported from these 17 genera, and about 53% of the isolated peptides showed cytotoxic, antibacterial and antiviral activities. In 2020, Jiang et al. reviewed the chemical structure and bioactive properties of new terpenes from marine derived fungi, as well as the biodiversity of these fungi from 2015 to 2019 [19]. *Penicillium*, *Aspergillus* and *Trichoderma* fungi were the main producers of terpenes. In 2021, Rani et al. reviewed the research status of microbial antibacterial molecules [10]. In 2022, Li et al., reviewed the chemistry and bioactivity of marine-derived bisabolane sesquiterpenoids [1]. In 2013, Lee et al. reviewed the bioactive secondary metabolites of *Aspergillus* derived from marine sources [42]. In 2018, Wang et al. reviewed 232 new bioactive metabolites from *Aspergillus* of marine origin from 2006 to 2016 and classified their bioactivity and chemical structures [43]. In 2020, Xu et al. reviewed the structural diversity and biological activity of 130 heterocyclic alkaloids produced by *Aspergillus* of marine origin from early 2014 to late 2018 [44]. However, there have been no studies on the antimicrobial compounds from marine *Aspergillus* in the last two years despite the fact that over the past two years, reports of antibacterial metabolites from *Aspergillus* have increased [45,46,47,48,49,50,51]. It is believed that the study of *Aspergillus* living in marine environments will facilitate the isolation of new fungal species and lead to the discovery of new compounds. Therefore, this review updates current compounds to cover metabolites isolated from marine *Aspergillus* between January 2021 and March 2023. It also provides structural diversity of compounds, as well as detailed information on sources and associated antimicrobial activity. We introduced the structural diversity and antimicrobial activity of 98 compounds isolated from marine-derived *Aspergillus*. This study will contribute to a better understanding of the chemical properties and biological activities of natural products from marine *Aspergillus*, thus facilitating drug discovery and development.

## 2. *Aspergillus* sp. from Various Marine Sources and Their Antimicrobial Activities

### 2.1. Aspergillus sp. from Marine Animals and Their Antimicrobial Activities

**Figure 1:**
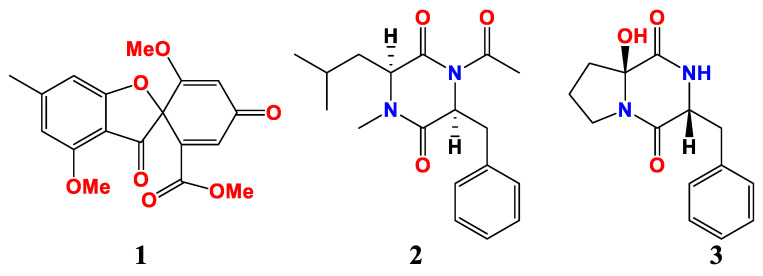
Compounds of *Aspergillus* sp. derived from marine animals.

Trypacidin (**1**) was isolated from the *A. fumigatus* HX-1 associated with clams (Figure 1). The anti-*Vibrio harveyi* activity of trypacidin was the same as that of streptomycin sulfate, and the minimum inhibitory concentration (MIC) was 31.25 μg/mL [52].

Two new dipeptides, asperopiperazines A and B (**2** and **3**), were obtained from *Aspergillus* sp. DY001 (Figure 1). The MICs of asperopiperazines A and B against *Escherichia coli* were 8 and 4 μM, and 8 and 8 μM against *S. aureus*, respectively [53].

In conclusion, only two *Aspergillus* species producing antimicrobial compounds are found from marine animals (except sponges and corals). Three compounds from these two *Aspergillus* strains have been reviewed for their antimicrobial activities. Notably, asperopiperazines A and B from *Aspergillus* sp. DY001 showed potent antimicrobial activities against *E. coli* and *S. aureus*.

### 2.2. Aspergillus sp. from Marine Plants and Their Antimicrobial Activities

**Figure 2:**
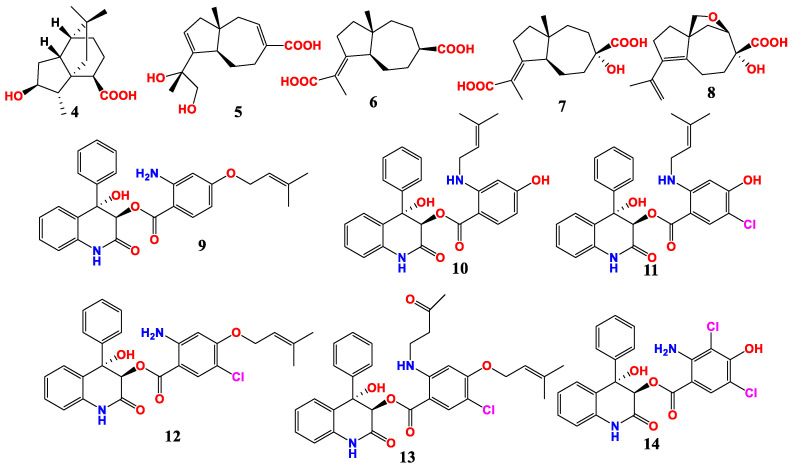
Compounds of *Aspergillus* sp. derived from marine plants.

Six new terpenoids were isolated from a seaward fungus *A. alabamensis* (Figure 2). They are asperalacids A-E and 4-hydroxy-5-(6)-dihydroterrecyclic acid A (**4**). Compound **4** and asperalacids A–D (**5**–**8**) showed antimicrobial activities against plant pathogenic fungi *Penicillium italicum*, *Fusarium graminearum* and *F. oxysporum*, as well as *S. aureus* and the Gram-positive bacteria *Bacillus subtilis*. Both MICs of asperalacids A and D against *F. graminearum* were 200 μg/mL. The MIC of asperalacids B and C against *F. oxysporum* were 100 and 100 μg/mL, and 200 and 25 μg/mL against *F. graminearum*, respectively. The MIC of compound **8** against *P. italicum*, *F. graminearum*, *F. oxysporum* and *S. aureus* were 200, 50, 100 and 25 μg/mL, respectively [54].

Eight new benzoic acid-containing alkaloids were isolated and identified from *A. alabamensis*. Among these compounds, asperalins A–F (**9**–**14**) showed moderate or strong inhibitory activities against some fish pathogens, *Streptococcus parauberis*, *S. iniae* and *Edwardsiella ictalurid* (Figure 2). Asperalins C and D showed strong antibacterial activities against *S. aureus*, *S. parauberis* and *S. iniae*, with MIC values of 10.1, 10.1 and 5.0 μM, respectively. Asperalin E had the strongest inhibitory effect on *S. iniae* with an MIC value of 2.2 μM. Notably, the MICs of asperalin F against four Gram-positive bacteria *S. aureus*, *B. subtilis*, *S. parauberis*, *S. iniae* and one Gram-negative bacterium *E. ictalurid* were 21.8, 87.3, 21.8, 43.6 and 10.9 μM, respectively [55].

In conclusion, *Aspergillus* species and its active metabolites from marine plant sources (except mangrove and seagrasses) were summarized. Eleven antimicrobial compounds were identified in the seagrass-derived fungus *A. alabamensis* during 2022 and 2023. Compounds **4**–**8** had a weak inhibitory effect on plant pathogens. However, compounds **11**–**14** showed strong antibacterial effects against *S. aureus*, *S. iniae* and some Gram-positive bacteria.

### 2.3. Aspergillus sp. from Mangroves and Their Antimicrobial Activities

**Figure 3:**
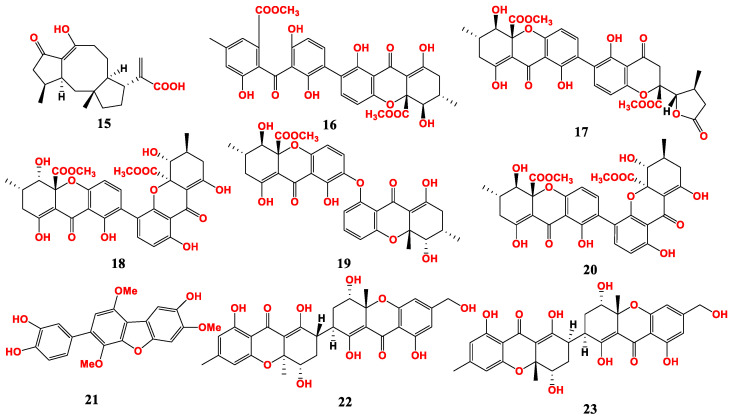
Compounds of *Aspergillus* sp. derived from mangroves.

Six antibacterial compounds were isolated from the marine fungus *A. brunneoviolaceus* MF180246 (Figure 3). These compounds included asperbrunneo acid (**15**), secalonic acid H (**16**), chrysoxanthone C (**17**), secalonic acid F1 (**18**), asperdichrome (**19**) and penicillixanthone A (**20**). They showed antibacterial activity against *S. aureus* with MIC values of 200, 50, 50, 25, 25 and 6.25 μg/mL [27].

Six polyhydroxy p-terphenyls (asperterphenyllins A–F) were isolated from the endophytic fungus *A. candidus* LDJ-5 in mangroves. Only asperterphenyllin C (**21**) showed antibacterial activity against *Proteus* sp. with an MIC value of 19 μg/mL [56].

Two new heterodimeric tetrahydroxanthones, aflaxanthones A and B (**22** and **23**), were isolated from *A. flavus* QQYZ. These two compounds showed potential antimicrobial activity and broad spectrum against several pathogenic fungi such as *C. albicans* and *F. oxysporum*, with MIC values in the range of 3.13–50 μM. They also showed moderate antibacterial activity against several bacteria such as *B. subtilis* and methicillin-resistant *S. aureus* (MRSA), with MIC values in the range of 12.5–25 μM [57].

In conclusion, *Aspergillus* and its active metabolites from mangroves were summarized. Due to the special geographical environment, mangroves had a wide variety of organisms, which has been thoroughly examined in previous studies of metabolites. Nine antimicrobial compounds were found in three *Aspergillus* strains from mangrove sources. Most of the compounds showed moderate antimicrobial activities. Among these compounds, compound 20 showed a strong inhibitory effect on *S. aureus*.

### 2.4. Aspergillus sp. Derived from Algae and Their Antimicrobial Activities

**Figure 4:**
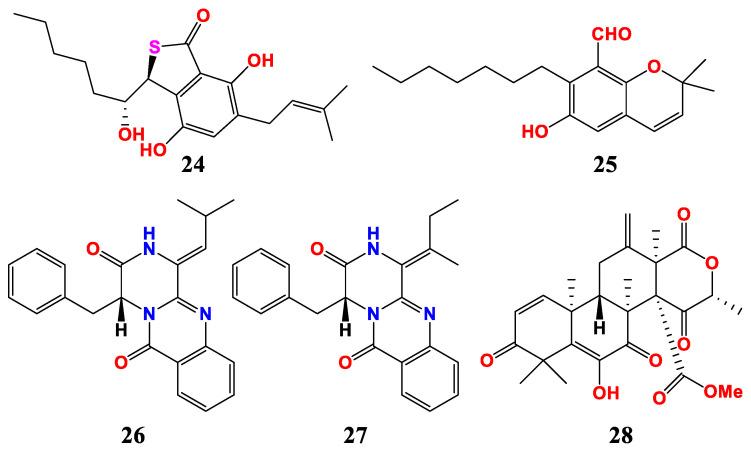
Compounds of *Aspergillus* sp. derived from algae.

Two C_7_-alkylated salicylaldehyde derivatives metabolites, namely asperglaucins A and B (**24** and **25**), were isolated from the endophytic fungus *A. chevalieri* SQ-8 (Figure 4). Asperglaucins A and B showed potent antimicrobial activities against plant pathogens *B. cereus* and *Pseudomonas syringae* pv *actinidae* (Psa), with an MIC value of 6.25 μM. Further analysis showed that asperglaucins A and B may change the external structure of *B. cereus* and Psa and cause cell membrane rupture or deformation. The results indicated that asperglaucins A and B may be potential lead compounds of pesticide fungicides [58].

Two new diketopiperazines, namely versiamide A (**26**) and 3, 15-dehydroprotuboxepin K (**27**), were isolated from endophytic fungus *A. creber* EN-602 obtained from the marine red algae *Rhodomela confervoides*. Versiamide A and 3, 15-dehydroprotuboxepin K showed inhibitory activities against a variety of aquatic bacteria, with MIC values ranging from 8 to 64 μg/mL. Versiamide A showed antibacterial activity against *Aeromonas hydrophila*, *E. coli*, *Micrococcus luteus* and *P. aeruginosa*, with MIC values of 64, 16, 64 and 64 μg/mL. 3, 15-dehydroprotuboxepin K showed antibacterial activity against *E. tarda*, *E. coli*, *M. luteus*, *P. aeruginosa* and *V. harveyi*, with MIC values of 64, 8, 16, 32 and 64 μg/mL [59].

An antibacterial terpenoid, namely terretonin F (**28**), were isolated from the *Aspergillus* sp. RR-YLW12, which derived from marine red algae *R. confervoide*. Terretonin F showed significant inhibitory activities against *Chattonella marina*, *Heterosigma akashiwo* and *Prorocentrum donghaiense*, with IC_50_ values of 3.1, 5.2 and 10.5 μg/mL, respectively [60].

In conclusion, *Aspergillus* species from marine algae and active metabolites were summarized. Five antimicrobial compounds were found in three fungi strains of algae origin. It should be noted that asperglaucins A and B (**24** and **25**) showed a strong inhibitory effect on *B. cereus*. The possible bacteriostatic mechanism of the compounds was also introduced. At present, the studies on the structure and biological activity of compounds are abundant, but the studies on the mechanism of biological activity are limited.

### 2.5. Aspergillus sp. from Corals and Their Antimicrobial Activities

**Figure 5:**
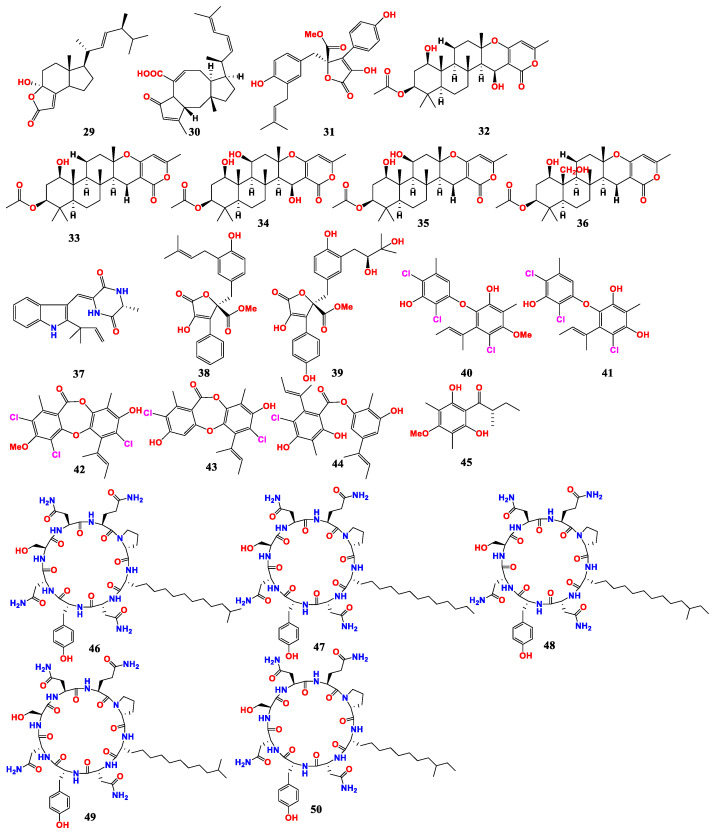
Compounds of *Aspergillus* sp. derived from corals.

Three known metabolites, including demethylincisterol A_2_ (**29**), asperophiobolin E (**30**) and butyrolactone I (**31**), were isolated and identified from the soft coral fungus *A. hiratsukae* SCSIO 5B_n1_003 (Figure 5). Compounds **29**–**31** showed potent antibacterial activity against *B. subtilis*, with MIC values of 10.26 ± 0.76, 17.00 ± 1.25 and 5.30 ± 0.29 μM. Meanwhile, asperophiobolin E and butyrolactone I showed weak activity against *S. aureus*, with MIC values of 102.86 ± 4.50 and 59.54 ± 0.50 μM, respectively [61].

Five new antimicrobial α-pyranone methterpenoids H-L (**32**–**36**) and one known antimicrobial compound, namely neoechinulin A (**37**), were isolated from *A. hiratsukae* SCSIO 7S2001, a fungus derived from ophiophora coral. Methterpenoids H-L and neoechinulin A showed varying degrees of antibacterial activity, with MIC values of 6.25–100 μg/mL. The MIC values of methterpenoid H were 6.25 μg/mL for *Micrococcus lutea* 01, MRSA, and *Streptococcus faecalis*; that of methterpenoid I was 6.25 μg/mL for MRSA; that of methterpenoid G was 12.5 μg/mL for MRSA; that of methterpenoid K was 6.25 μg/mL for *Klebsiella pneumoniae*; that of methterpenoid L was 12.5 μg/mL for *M. lutea*, *S. faecalis* and MRSA; and that of neoechinulin A was 12.5 μg/mL for *S. faecalis*. [62].

Two butenolides, including versicolactone B (**38**) and butyrolactone VI (**39**), were isolated from *Aspergillus terreus* SCSIO41404, a fungus derived from coral. Versicolactone B and butyrolactone VI showed weak antibacterial activity against *Enterococcus faecalis* and *K. pneumoniae* with IC_50_ values of 25 and 50 μg/mL, respectively [63].

Six chlorinated polyketones were isolated from the coral fungus *A. unguis* GXIMD 02505 in the Beibu Gulf. These polyketones included aspergillusethers J and F (**40** and **41**), nornidulin (**42**), aspergillusidones B and C (**43** and **44**) and 1-(2, 6-dihydroxy-4-methoxy-3, 5-dimethylphenyl)- 2-methylbutan-1-one (**45**). Compounds **40**–**45** exhibited inhibitory activities against marine biofilm-forming bacteria, *Marinobacterium jannaschii*, MRSA, *Microbulbifer variabilis* and *Vibrio pelagius*, with MIC values ranging from 2 to 64 μg/mL [64].

Five antimicrobial cyclic lipopeptides, namely maribasins C-E (**46**–**48**) and maribasins A and B (**49** and **50**), were isolated from the marine fungus *Aspergillus* sp. SCSIO 41501. These compounds showed strong antifungal activities against five plant pathogenic fungi, with MIC values ranging from 3.12 to 50 μg/disc [34].

In conclusion, coral-derived *Aspergillus* and its active metabolites were summarized. Twenty-two antimicrobial compounds were found in five fungi strains of coral origin. It was a relatively large variety of compounds compared with *Aspergillus* from other origins. Most of the compounds had a wide antimicrobial spectrum against different bacteria and fungi.

### 2.6. Aspergillus sp. Derived from Sponges and Their Antimicrobial Activities

**Figure 6:**
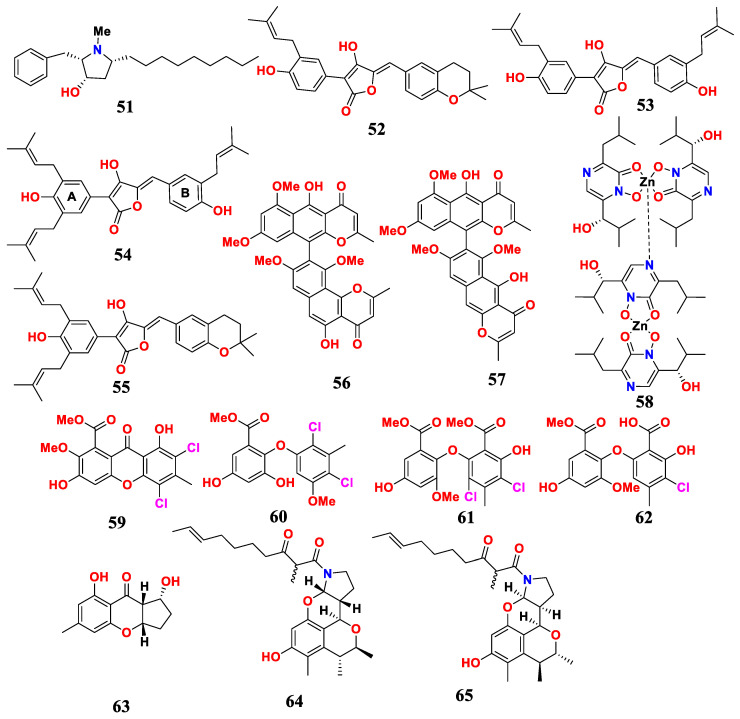
Compounds of *Aspergillus* sp. derived from sponges.

One hydroxypyrrolidine alkaloid preussin (**51**) was isolated and identified from marine sponge-related fungus *A. candius* KUFA 0062 (Figure 6). Preussin showed inhibition against vancomycin-resistant *Enterococcus* (VRE) and MRSA, as well as *E. faecalis* ATCC29212 and *S. aureus* ATCC 29213 [65].

Four antimicrobial compounds were isolated from the marine sponge-derived fungus *Aspergillu flavus* KUFA1152. These compounds were aspulvinones B’, H, R and S (**52**–**55**). Aspulvinones B’, H, R and S showed antibacterial activity against some multidrug-resistant strains isolated from the environment, and inhibited the biofilm formation of strains. Aspulvinones B’ and H displayed activity with MIC values of 16 μg/mL for the *S. aureus*, and for *E. faecalis*, MIC values ranged from 16 to 64 μg/mL. Aspulvinones R and S exhibited the potent activity against all Gram-positive strains tested, with MIC values ranging from 4 to 16 μg/mL for *S. aureus* and *E. faecalis*, and from 8 to 16 μg/mL for the VRE and MRSA [66].

The endophytic fungus *A. niger* L14 has been chemically studied, and two dimers, naphtho-γ-pyrone, fonsecinone A (**56**) and isoaurasperone A (**57**), have been isolated. These compounds had obvious inhibitory effects on human pathogenic bacteria *Helicobacter pylori* 159 and G27 with MIC values ≤ 4 μg/mL, comparable to the antibacterial effect of ampicillin sodium [67].

One antimicrobial compound, namely dizinc hydroxy-neotriamycin (**58**), was isolated from the sponge-related fungus *A. ochraceopetaliformis* SCSIO 41018. Dizinchydroxyneoaspergillin showed potent inhibition against MRSA, *Acinetobacter baumannii*, *E. faecalis*, *Staphyloccocus aureus* and *Klebsiella pneumonia*, with MIC values ranging from 0.45 to 7.8 μg/mL [68].

Two new chlorinated biphenyls, including aspergetherins A and C (**59** and **60**), and two known biphenyl derivatives, including methyl 3, 5-dichloroasterric acid (**61**) and methyl chloroasterrate (**62**), were isolated from a marine sponge symbiotic fungus *A. terreus* 164018. The antibacterial activity of these compounds against MRSA was evaluated, with MIC values ranging from 1.0 to 128 μg/mL. Notably, compound **61** had obvious inhibitory effects on two different MRSA strains, with MIC values of 1 and 16 μg/mL [69].

Chemical studies of the natural compounds of the marine fungus *Aspergillus* sp. LS57 had resulted in the isolation of aspergilluone A (**63**). The MIC value of aspergilluone A was 32 μg/mL against *Mycobacterium tuberculosis*, 64 μg/mL against *S. aureus*, and 128 μg/mL against both Gram-positive *B. subtilis* and Gram-negative *E. coli* [70].

Two novel tetracyclic skeleton alkaloids were isolated from *Aspergillus* sp. LS116, which were perinadines B and C (**64** and **65**). Perinadines B and C showed moderate antibacterial activity for *B. subtilis* with MIC values of 32 and 64 μg/mL [71].

In conclusion, *Aspergillus* and its active metabolites of sponge were summarized in this paper. Sponges are the most primitive marine animals with a large number of microorganisms, which are important sources of active natural products. Fifteen antibacterial compounds were found in seven fungi strains derived from sponge. *Aspergillus* derived from sponge was the source of antimicrobial compounds. Most of the compounds had a wide antimicrobial spectrum against a variety of bacteria and fungi. Hydroxy-neotriamycin (**58**) had a strong bacteriostatic effect on a variety of bacterial pathogens.

### 2.7. Aspergillus sp. from Seawater and Their Antimicrobial Activities

**Figure 7:**
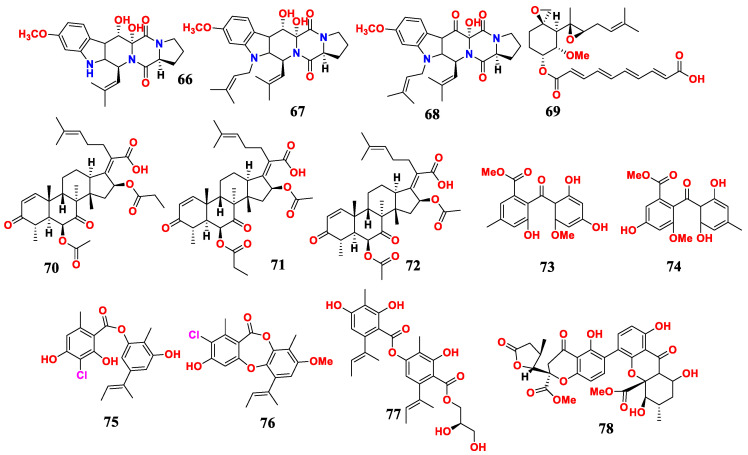
Compounds of *Aspergillus* sp. derived from seawater.

Nine antimicrobial compounds were isolated from marine fungus *A. fumigatus* H22. These compounds included 12,13-dihydroxyfumitremorgin C (**66**), fumitremorgin B (**67**), 13-oxofumitremorgin B (**68**), fumagillin (**69**), helvolic acid (**70**), 6-O-propionyl-16-O-deacetylhelvolic acid (**71**), 16-O-propionyl-6-O-deacetylhelvolic acid (**72**), penibenzophenone E (**73**) and sulochrin (**74**) (Figure 7). Compounds **66** and **68** showed potent antibacterial activity, and **69**–**74** exhibited strong anti-MRSA activity with MIC values between 1.25 and 2.5 μM. Additionally, compound **66** showed moderate inhibitory activity against *Mycobacterium Bovis*, with an MIC value of 25 μM, and compound **67** showed moderate inhibitory activity against *C. albicans*, with an MIC value of 50 μM [72].

Three novel phenolic polyketones, namely unguidepside C (**75**), aspersidone B (**76**) and agonodepside C (**77**), were isolated from *A. unguis*. These compounds showed a strong activity against Gram-positive bacteria, with MIC ranging from 5.3 to 22.1 μM [73].

Five novel dimeric tetrahydroxanthones, including aculeaxanthones A-E, were extracted from the marine fungus *A. aculeatinus* WHUF0198. Among them, only aculeaxanthone A (**78**) showed activity against *B. subtilis* 168, *S. aureus* USA300, *H. pylori* 159, *H. pylori* 129, *H. pylori* 26695 and *H. pylori* G27, with MIC values of 1.0, 2.0, 2.0, 2.0, 4.0 and 4.0 μg/mL, respectively [74].

In conclusion, *Aspergillus* and its active metabolites from seawater were summarized. Thirteen antimicrobial compounds were found in three fungi strains derived from seawater. Compounds **69**–**74** exhibited strong anti-MRSA activity and aculeaxanthone A (**78**) showed strong anti-bacterial pathogen activity.

### 2.8. Aspergillus sp. from Marine Sediments and Their Antimicrobial Activities

**Figure 8:**
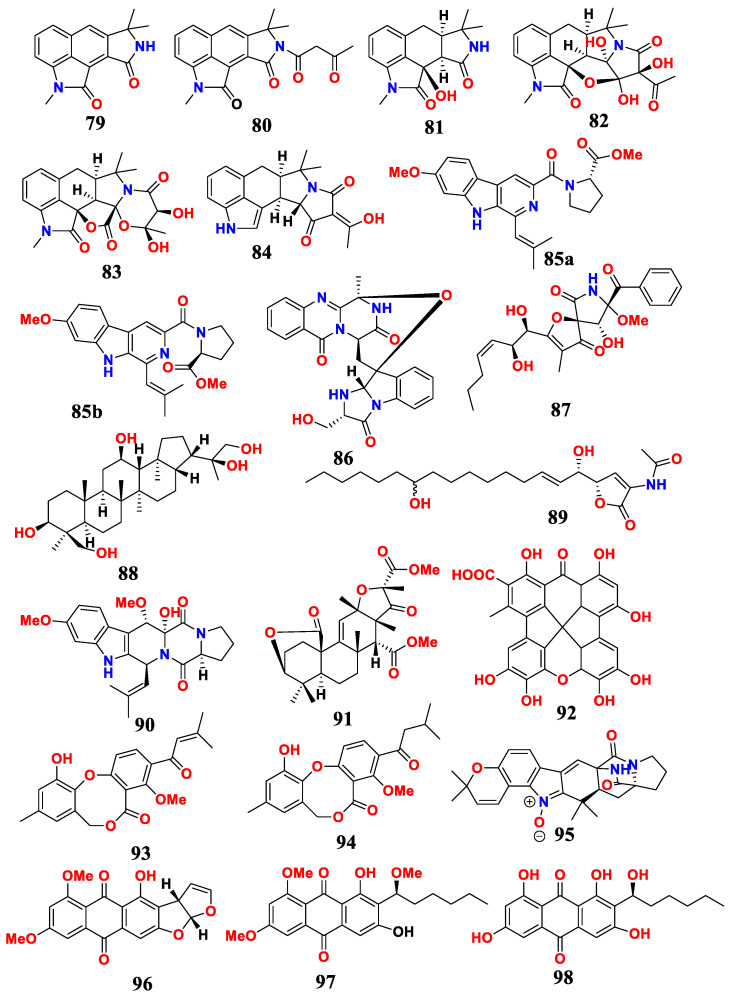
Compounds of *Aspergillus* sp. derived from marine sediments.

Six known compounds, including cyclopiamide (**79**), speradine H (**80**), speradine G (**81**), speradine B (**82**), speradine C (**83**) and cyclopiazonic acid (CPA) (**84**), were isolated from *A. flavus* SCSIO F025 from deep-sea sediments in the South China Sea (Figure 8). Compounds **79**–**84** showed weak antibacterial activity against *E. coli*, and CPA also exhibited strong antibacterial activity against MRSA, *B. subtilis*, *S. aureus*, *M. luteus* and *Bacillus thuringiensis* [75].

Five novel antibacterial metabolites and one known antibacterial compound were all isolated from the deep-sea sediment-derived fungus *A. fumigatus* SD-406. The novel metabolites included secofumitremorgins A and B (**85a** and **85b**), 29-hydroxyfumiquinazoline C (**86**), 10*R*-15-methylpseurotin A (**87**), 1,4,23-trihydroxy-hopan-22,30-diol (**88**) and sphingofungin I (**89**), and one known cyclotryprostatin B (**90**). Compounds **85**–**90** exhibited inhibitory activities against pathogenic bacteria and plant pathogenic fungi, with MIC values of 4–64 μg/mL [76].

One new metabolite, namely 3, 5-dimethylorsellinic acid-based meroterpenoid (**91**), was isolated from the deep-sea fungus *Aspergillus* sp. CSYZ-1. Compound **91** showed strong antimicrobial activity against *S. aureus* and *H. pylori*, with MIC values of 2–16 and 1–4 μg/mL, respectively [77].

Two novel antibacterial metabolites, including aspergiloxathene A (**92**) and Δ^2′^-1′-dehydropenicillide (**93**) and one known antibacterial compound, namely dehydropenicillide (**94**), were isolated from *Aspergillus* sp. IMCASMF180035. Aspergiloxathene A exhibited significant inhibition against MRSA and *S. aureus*, with MIC values of 22.40 and 5.60 μM. Dehydropenicillide and Δ^2′^-1′-dehydropenicillide showed potent antibacterial activities against *H. pylori*, with MIC values of 21.61 and 21.73 μM, respectively [30].

One alkaloid asperthrin A (**95**) had been isolated from the marine endophytic fungus *Aspergillus* sp. YJ191021. The isolated compound had inhibitory effects on *Rhizoctonia solani*, *Xanthomonas oryzae* pv. *Oryzicola* and *Vibrio anguillarum*, with MIC values of 25, 12.5 and 8 μg/mL, respectively [78].

Three antimicrobial compounds were isolated from the fermented extracts of *Aspergillus* sp. WHUF05236. They included 6,8-di-O-methylversicolorin A (**96**), 6,8,1′-tri-O-methylaverantin (**97**) and 6,8-di-O-methylaverantin (**98**). They exhibited antibacterial activity against *H. pylori*, with MIC values ranging from 20.00 to 43.47 μM [79].

In conclusion, *Aspergillus* and its active metabolites from marine sediments were summarized. Twenty antimicrobial compounds were found in six *Aspergillus* strains from marine sediments. According to the literature, more than fifty antimicrobial compounds were produced by *Aspergillus* from marine sediments between 2018 and 2020. Therefore, marine sediments are an important source of secondary metabolites of fungi. Among them, compound **91** showed strong antimicrobial activity against *S. aureus* and *H. pylori*.

Sources and activities of compounds from marine *Aspergillus* were summarized in Table 1. We classified fungi and compounds according to *Aspergillus* origin.

**Figure 9:**
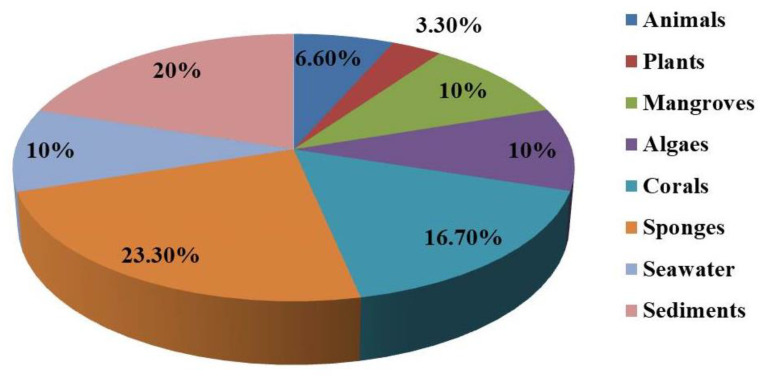
The proportion of Aspergillus from different marine sources.

In recent years, marine fungi have attracted the attention of researchers due to their bioactive compounds [10,44,46,80,81,82,83,84,85]. Combined with a series of previous excellent literature reviews, we conducted a comprehensive literature review of antibacterial compounds produced by Aspergillus fungi of different marine origin during the period of 2021–2023. The reported numbers of *Aspergillus* from marine animals, plants, mangroves, seagrasses, coral, sponge, seawater and marine sediment are shown in Figure 9. The most *Aspergillus* was derived from sponges, accounting for 23.30%. *Aspergillus* derived from marine coral was found in the second place, accounting for 16.7%.

**Figure 10:**
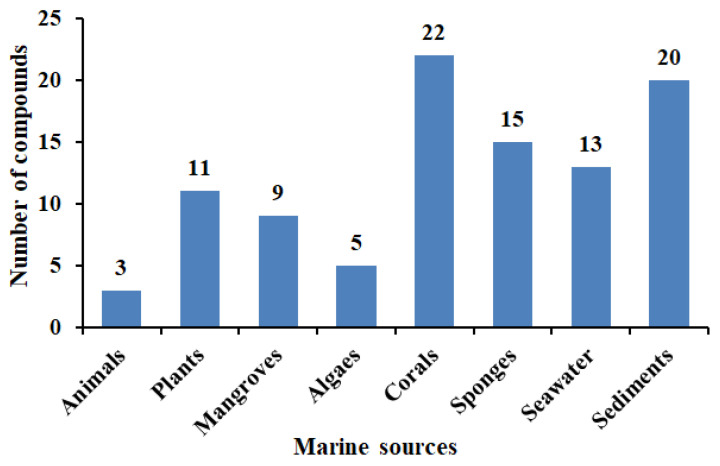
The proportion of *Aspergillus* compounds from different marine sources.

We summarized ninety-eight antibacterial compounds from *Aspergillus* strains isolated from different marine sources (Figure 10). Among them, twenty-two antimicrobial compounds were found in marine corals from January 2021 to March 2023. Marine sediments had the next highest number of antimicrobial compounds, with twenty compounds. Therefore, in recent years, the antimicrobial compounds of *Aspergillus* from marine sources mainly came from marine corals and marine sediments. Marine natural products are rich in species and play an obvious role in the treatment of pathogen infections [86,87,88,89,90,91,92]. More and more novel compounds with different chemical structures and biological activities are being discovered [48,93,94,95,96,97,98,99].

## 3. Conclusions

This review describes antimicrobial compounds from *Aspergillus* species during January 2021 to March 2023. Ninety-eight compounds derived from *Aspergillus* species were described. Only three compounds with antimicrobial activities are found from marine animals (except sponges and corals). Twenty-two antimicrobial compounds were found in five fungi strains of coral origin. Fifteen antibacterial compounds were found in seven fungi strains derived from sponge. Most of these thirty-seven compounds had a wide antimicrobial spectrum against a variety of bacteria and fungi. Except for the compounds derived from coral and sponge, most of the compounds from other sources showed antibacterial activity, but no fungal inhibitory activity. Most of the compounds had inhibitory effects on *S. aureus*. Some compounds exhibited inhibitory effects on *E. coli* and *B. subtilis.* Among them, compound 91 showed strong antimicrobial activity against *H. pylori*. These active compounds have potential applications in bacterial and fungal infections and will provide reference for the development of novel anti-infective drugs.

## Figures and Tables

**Table 1:** Sources and activities of compounds from marine *Aspergillus*.

Sources and *Aspergillus*	Compounds	Activities	References
Marine animals			
*A. fumigatus* HX-1	Trypacidin (**1**)	MIC (anti-*V. harveyi*) was 31.25 μg/mL	[52]
*Aspergillus* sp. DY001	Asperopiperazines A, B (**2**, **3**)	MIC (anti-*E. coli*) were 8 and 4 μM MIC (anti-*S. aureus*) were 8 and 8 μM	[53]
Marine plants			
*A. alabamensis*	4-hydroxy-5(6)-dihydroterrecyclic acid A (**4**), asperalacids A–D (**5**–**8**)	MIC (anti-plant pathogens) was 25–200 μg/mL	[54]
*A. alabamensis*	asperalins A–F (**9**–**14**)	MIC (anti-fish pathogens) was 2.2–87.3 μM	[55]
Mangroves			
*A. brunneoviolaceus* MF180246	asperbrunneo acid (**15**), secalonic acids H, F1 (**16**, **18**), chrysoxanthone C (**17**), asperdichrome (**19**), penicillixanthone A (**20**)	MIC (anti-*S. aureus*) were 200, 50, 50, 25, 25, 6.25 μg/mL	[27]
*A. candius* LDJ-5	asperterphenyllin C (**21**)	MIC (anti-*Proteus* sp.) was 19 μg/mL	[56]
*A. flavus* QQYZ	aflatoxones A, B (**22**, **23**)	MIC (anti-pathogens) was 3.13–50 μM	[57]
Marine algaes			
*A. chevalieri* SQ-8	asperglaucins A, B (**24**, **25**)	MIC (anti-plant pathogens) was 6.25 μM	[58]
*A. creber* EN-602	versiamide A (**26**), 3, 15-dehydroprotuboxepin K (**27**)	MIC (anti-bacteria) was 8–64 μg/mL	[59]
*Aspergillus* sp. RR-YLW12	terretonin F (**28**)	IC_50_ (anti-three microalgae) were 3.1, 5.2, 10.5 μg/mL	[60]
Marine corals			
*A. hiratsukae* SCSIO 5B_n1_003	demethylincisterol A_2_ (**29**), asperophiobolin E (**30**), butyrolactone I (**31**)	MIC (anti-*B. subtilis*) were 10.26 ± 0.76, 17.00 ± 1.25 and 5.30 ± 0.29 μM	[61]
*A. hiratsukae* SCSIO 7S2001	methterpenoids H-L (**32**–**36**) neoechinulin A (**37**)	MIC (anti-bacteria) was 6.25–100 μg/mL	[62]
*A. terreus* SCSIO41404	versicolactone B (**38**), butyrolactone VI (**39**)	IC_50_ (anti-*E. faecalis*, *K. pneumoniae*) were 25 and 50 μg/mL	[63]
*A. unguis* GXIMD 02505	**40**–**45**	MIC (anti-bacteria) was 2–64 μg/mL	[64]
*Aspergillus* sp. SCSIO 41501	maribasins C–E,A,B (**46**–**50**)	MIC (anti-plant pathogens) was 3.12–50 μg/disc	[34]
Sponges			
*A. candius* KUFA 0062	preussin (**51**)	anti-pathogens	[65]
*A. flavipes* KUFA1152	aspulvinones B’, H, R and S (**52**–**55**)	MIC (anti-pathogens) was 16–64 μg/mL	[66]
*A. niger* L14	fonsecinone A (**56**), isoaurasperone A (**57**)	MIC (anti-*H. pylori*) was ≤4 μg/mL	[67]
*A. ochraceopetaliformis* SCSIO 41018	hydroxy-neotriamycin (**58**)	MIC (anti-pathogens) was 0.45–7.8 μg/mL μM	[68]
*A. terreus* 164018	aspergetherins A, C (**59**, **60**) 3, 5-dichloroasterric acid (**61**), methyl chloroasterrate (**62**)	MIC (anti-MRSA) was 1.0–128 μg/mL	[69]
*Aspergillus* sp. LS57	aspergilluone A (**63**)	MIC (anti-pathogens) was 32–128 μg/mL	[70]
*Aspergillus* sp. LS116	perinadines B, C (**64**, **65**)	MIC (anti-*B. subtilis*) were 32 and 64 μg/mL	[71]
Seawater			
*A. fumigatus* H22	12,13-dihydroxyfumitremorgin C (**66**), fumitremorgin B (**67**)	MIC(anti-*M. Bovis*, *C. albicans*) were 25 and 50 μM	[72]
*A. fumigatus* H22	(**66**),13-oxofumitremorgin B (**68**)	antibacterial activity	[72]
*A. fumigatus* H22	fumagillin (**69**), helvolic acid (**70**), 6-O-propionyl-16-O-deacetylhelvolic acid (**71**), 16-O-propionyl-6-O-deacetylhelvolic acid (**72**), penibenzophenone E (**73**), sulochrin (**74**)	MIC (anti-MRSA) were 1.25 and 2.5	[72]
*A. unguis*	unguidepside C (**75**), aspersidone B (**76**), agonodepside C (**77**)	MIC (anti-bacteria) was 5.3 to 22.1 μM	[73]
*A. aculeatinus* WHUF0198	aculeaxanthone A (**78**)	MIC (anti-bacteria) was 1.0 to 4.0 μM	[74]
Marine sediments			
*A. flavus* SCSIO F025	cyclopiamide (**79**), speradines G,H,B,C (**80**–**83**), CPA (**84**)	weak anti-bacteria	[75]
*A. fumigatus* SD-406	**85**–**90**	MIC (anti-bacteria and plant pathogens) were 4–64 μg/mL	[76]
*Aspergillus* sp. CSYZ-1	meroterpenoid (**91**)	MIC (anti-*S. aureus*, *H. pylori*) were 2–16 and 1–4 μg/mL	[77]
*Aspergillus* sp. IMCASMF180035	aspergiloxathene A (**92**)	MIC (anti-MRSA, *S. aureus*) were 22.40 and 5.60 μM	[30]
*Aspergillus* sp. IMCASMF180035	Δ^2′^-1′-dehydropenicillide (**93**), dehydropenicillide (**94**)	MIC (anti-*H. pylori*) were 21.61 and 21.73 μM	[30]
*Aspergillus* sp. YJ191021	asperthrins A (**95**)	MIC (anti-plant pathogens) was 8–25μg/mL	[78]
*Aspergillus* sp. WHUF05236	6, 8-di-O-methylversicolorin A (**96**), 6,8,1′-tri-O-methylaverantin (**97**), 6,8-di-O-methylaverantin (**98**)	MIC (anti-*H. pylori*) was 20.00 to 43.47 μM	[79]

## Data Availability

Not applicable.
